# Knock-in Luciferase Reporter Mice for *In Vivo* Monitoring of CREB Activity

**DOI:** 10.1371/journal.pone.0158274

**Published:** 2016-06-23

**Authors:** Dmitry Akhmedov, Kavitha Rajendran, Maria G. Mendoza-Rodriguez, Rebecca Berdeaux

**Affiliations:** 1 Department of Integrative Biology and Pharmacology, McGovern Medical School at the University of Texas Health Science Center at Houston (UTHealth), Houston, Texas, United States of America; 2 Center for Metabolic and Degenerative Diseases, Institute of Molecular Medicine, University of Texas Health Science Center at Houston (UTHealth), Houston, Texas, United States of America; 3 Cell and Regulatory Biology Program, The University of Texas Graduate School of Biomedical Sciences at Houston, McGovern Medical School at the University of Texas Health Science Center at Houston (UTHealth), Houston, Texas, United States of America; The Roslin Institute, UNITED KINGDOM

## Abstract

The cAMP response element binding protein (CREB) is induced during fasting in the liver, where it stimulates transcription of rate-limiting gluconeogenic genes to maintain metabolic homeostasis. Adenoviral and transgenic CREB reporters have been used to monitor hepatic CREB activity non-invasively using bioluminescence reporter imaging. However, adenoviral vectors and randomly inserted transgenes have several limitations. To overcome disadvantages of the currently used strategies, we created a *ROSA26* knock-in CREB reporter mouse line (*ROSA26-CRE-luc)*. cAMP-inducing ligands stimulate the reporter in primary hepatocytes and myocytes from *ROSA26-CRE-luc* animals. *In vivo*, these animals exhibit little hepatic CREB activity in the *ad libitum* fed state but robust induction after fasting. Strikingly, CREB was markedly stimulated in liver, but not in skeletal muscle, after overnight voluntary wheel-running exercise, uncovering differential regulation of CREB in these tissues under catabolic states. The *ROSA26-CRE-luc* mouse line is a useful resource to study dynamics of CREB activity longitudinally *in vivo* and can be used as a source of primary cells for analysis of CREB regulatory pathways *ex vivo*.

## Introduction

The cAMP response element binding protein (CREB) is a key transcription factor in the response to endocrine hormones that stimulate G-protein coupled receptors to initiate cAMP signaling [1]. CREB binds directly to cAMP response elements (CRE) on the proximal promoter regions of target genes and recruits co-activators CREB binding protein (CBP) and CREB-regulated transcriptional co-activators (CRTCs) to form a ternary complex in response to cAMP/PKA signaling. During fasting, glucagon stimulates CREB transcriptional complex activity that is critical for stimulation of gluconeogenic genes, as mice lacking CREB or CRTC2 activity in liver have defective initiation of gluconeogenic gene transcription upon fasting [2–5]. The significance of this pathway to metabolic homeostasis is underscored by the finding that CREB/CRTC2 activity is highly activated in obese mice, and strategies to inhibit CREB or CRTC2 ameliorate hyperglycemia in obese diabetic animals [3, 6–8].

Because CREB is dynamically regulated by nutritional state as well as the phase of the circadian cycle [1, 9, 10], a bioluminescence reporter strategy has been developed to enable monitoring of CREB transcriptional activity in living mice *in vivo*. An adenoviral vector encoding multimerized cAMP response elements in the context of a minimal CFTR promoter driving firefly luciferase enabled visualization of hepatic CREB activity in living mice after fasting, other genetic modifications, or acute knock-down or over-expression of CREB regulatory proteins [8–12]. This tool provided important insights into CREB regulation. However, disadvantages of adenoviral use *in vivo* such as restricted tropism to liver, short-lived expression of adenovirally-expressed genes, and inflammation [13], as well as the requirement for post-hoc normalization using a co-injected control adenoviral vector [8], limit the utility of this strategy. To circumvent these challenges, we previously created a CREB-luciferase transgenic reporter mouse by randomly inserting into the mouse genome the same sequences from the adenoviral reporter [14]. Although useful for visualizing CREB activity in additional tissues, such as brown adipose [14], the traditional transgenic approach presented different constraints, including silencing of the transgene over generations in some founder lines, unknown copy number and unknown insertion site. Moreover, both of these strategies employed original firefly luciferase, the sequence of which is not optimized for expression in mammalian cells.

To facilitate longitudinal monitoring of CREB activity in multiple tissues of individual animals without concerns about adenovirus, random transgene insertion or weak luciferase signal due to codon usage, we generated a *ROSA26* knock-in mouse with a single copy of a CREB-sensitive, codon-optimized, destabilized luciferase transgene ("CRE-luc”). Similar to other *in vivo* CREB reporters, we observed induction of hepatic and brain CREB activity in response to fasting. We also observed robust CREB activation in liver and brain after voluntary exercise. Our results describe a knock-in reporter allele that will be useful for *in vivo* monitoring of CREB activity in living animals in longitudinal studies as well as for cell-based assays.

## Materials and Methods

### Ethics statement

This study was carried out in strict accordance with the recommendations in the Guide for the Care and Use of Laboratory Animals of the National Institutes of Health. The protocols for animal studies were approved by the Animal Welfare Committee (IACUC) of the McGovern Medical School at the University of Texas Health Science Center Houston (permit numbers: AWC-11-094, AWC-11-095, AWC-11-096, AWC-14-0071). All efforts were made to minimize suffering or distress. For imaging studies, animals were anesthetized with inhaled isoflurane in oxygen. Euthanasia methods were exsanguination under isoflurane anesthesia, decapitation into liquid N_2_ (neonates) or CO_2_ overdose under CO_2_ anesthesia.

### Generation of *ROSA26-CRE-luc* reporter mice

A fragment containing the CRE-*Luc2P*-SV40 poly(A) cassette was excised from pGL4.29 plasmid (Promega, Genbank: DQ904461.1) using *BamHI* /*SpeI*, cloned into *BamHI*/ *SpeI* sites of a modified pBluescript vector containing two *XmaI* sites (pBS-Xma2) and then sub-cloned into the Ai9-tdTomato vector [15] using *XmaI* sites. This insert contains two full (-171 and -113) and two half (-145 and -135) CREB binding sites (cAMP response elements, CRE) and a minimal promoter containing a TATA box (-57), *Luc2P* (codon optimized firefly luciferase (*Luc2*) fused to a PEST domain to enhance turnover) and the SV40 late poly(A) polyadenylation signal. The resulting targeting construct pAi9-CRE-luc was confirmed by sequencing, linearized with *SgrDI*, purified and electroporated into mouse 129/SvImJ ES cells. Clones were screened by Southern blotting with 5’ and 3’ probes after DNA digestion with *EcoRI* and *EcoRV*, respectively. Mouse ES work, clone re-growth and clone injection were performed by the Mouse ES Cell Core and Genetically Engineered Mouse Core at Baylor College of Medicine, Houston, TX. Chimeric male mice were bred with albino female *C57BL/6J-Tyr*^*c-2J*^*/J* (“albino Bl6”) (Jackson). Agouti pups containing the transgene were used as founders. Animals were back-crossed to albino Bl6 for at least 3 generations; albino transgenic animals were chosen for further breeding. For an unknown reason, up to 10% of *ROSA26-CRE-luc* mice never show substantial bioluminescence signals in any tissue and/or do not respond to fasting; non-responders are excluded from breeding and experimental cohorts. The strain is termed *Gt(ROSA)26Sor*^*tm2(CAG-tdTomato*,*cAMPRE-luc)Berd*^ (“*ROSA26-CRE-luc*”), MGI 5696733. Primer sequences are reported in S1 Table.

### *In vivo* bioluminescence imaging

Bioluminescence imaging was performed as described [8] on isoflurane-anesthetized animals injected IP with 100 mg/kg D-luciferin (122799, Perkin Elmer) in sterile 0.9% saline (USP). Briefly, animals were placed on a heated stage (37°C) of an IVIS Lumina XR imager (Caliper Life Sciences) equipped with an isoflurane manifold for continuous anesthesia. Two to five minutes after D-luciferin injection, a monochrome photograph was acquired followed immediately by bioluminescence acquisition, with multiple exposures from 5–40 sec. Instrument settings were at f-stop 1, binning 4. Total luminescence flux (radiance in photons/s) from a region of interest over the liver or the ventral region of the skull was quantified on overlaid photographic and bioluminescence image files using Living Image 4.4 software (Caliper Life Sciences) on exposure-matched images (5–40 sec) within an experiment. Bioluminescence images were exported as false-colored images using matched visualization scales.

### Mouse experiments

Animals were housed at 22°C in individually ventilated cages with a 12 h light/dark cycle (9AM-9PM for fasting studies; 7AM-7PM for exercise studies) with free access to water and irradiated chow diet (LabDiet 5053). Male animals aged 8–20 weeks were used for fasting experiments because C57Bl/6 males have higher fasting glucose than females [16]; females aged 14 weeks were used for exercise experiments because females run more on voluntary wheels and are active for a longer duration than males [17 and our unpublished observations]. *Ad libitum* or basal images were taken the same day of fasting or exercise. Mice were fasted in cages with synthetic bedding at 5 PM (for 16 h fast, ZT8-day 2 ZT0) or 9 AM (for 6 h fast, ZT0-ZT6). In some experiments, animals were administered glucagon (100 μg/kg, Sigma G2044) in USP saline IP after overnight fast. For exercise experiments, animals were not pre-trained to run. Baseline images of female mice in static cages were obtained at 5 PM (ZT10) on the day of the exercise experiment and mice were allowed to recover from anesthesia in home cages. From lights out to lights on (ZT12-day2 ZT0), mice were placed in cages containing voluntary running wheels fitted with electronic monitors for activity tracking by Activity Wheel Monitor software (Lafayette Instruments). An “exercised” image was taken promptly at lights on; animals were euthanized immediately after imaging under isoflurane anesthesia for tissue collection.

### Primary cells

Primary hepatocytes were prepared from anesthetized mice by hepatic perfusion with type IV collagenase (Sigma, C5138, 120 U/mL) as described [18] and analyzed within 24 h of harvest. Primary myoblasts were harvested from neonatal mice by collagenase digestion and pre-plating, as described [19] and passaged up to 3 times. Cells were treated with glucagon (100 nM) or FSK/ IBMX (forskolin 10 μM/ isobutylmethylxanthine 18 μM; Sigma F6886, I5879) in triplicate and processed for luciferase assays using approximately 15 μg total cell lysate as described [18]. Luminescence (arbitrary units) was normalized to total protein, expressed as A.U./μg protein or fold change from un-stimulated cells.

### Analysis of liver and muscle tissue

Livers, quadriceps and gastrocnemius muscles were pulverized under LN_2_ and subsequently homogenized using a rotor-stator homogenizer in modified RIPA-T buffer containing 0.1% SDS and protease and phosphatase inhibitors as described [20]. Proteins were resolved using PAGE and blotted on PVDF membranes. Western blots were performed using antibodies against pCREB (#9198) or CREB (#9197) (Cell Signaling Technologies). For luciferase assay, liver extracts were prepared and analyzed as described [21]. RNA was isolated from liver using Aurum total RNA fatty and fibrous tissue kit (Bio-Rad). cDNA was prepared with Protoscript II reverse transcriptase (New England Biolabs) and oligo-dT primers. Gene expression was analyzed by real-time PCR using a LightCycler 480 (Roche Diagnostics GmbH) and gene specific primers (S1 Table), normalized to *Gapdh* internal control as described [20].

### Statistical analysis

For analysis of bioluminescence data, we used paired two-tailed Student’s *t*-test or Wilcoxon rank-sum tests (for non-normally distributed data) using GraphPad Prism6. For analysis of biochemical luciferase assays, CREB phosphorylation and mRNA expression data, we used unpaired two-tailed Student’s *t*-test. Correlation analysis was performed using the Pearson test in GraphPad Prism6.

## Results

### *ROSA26-CRE-luc* mice allow monitoring CREB activity during fasting

**Fig 1:**
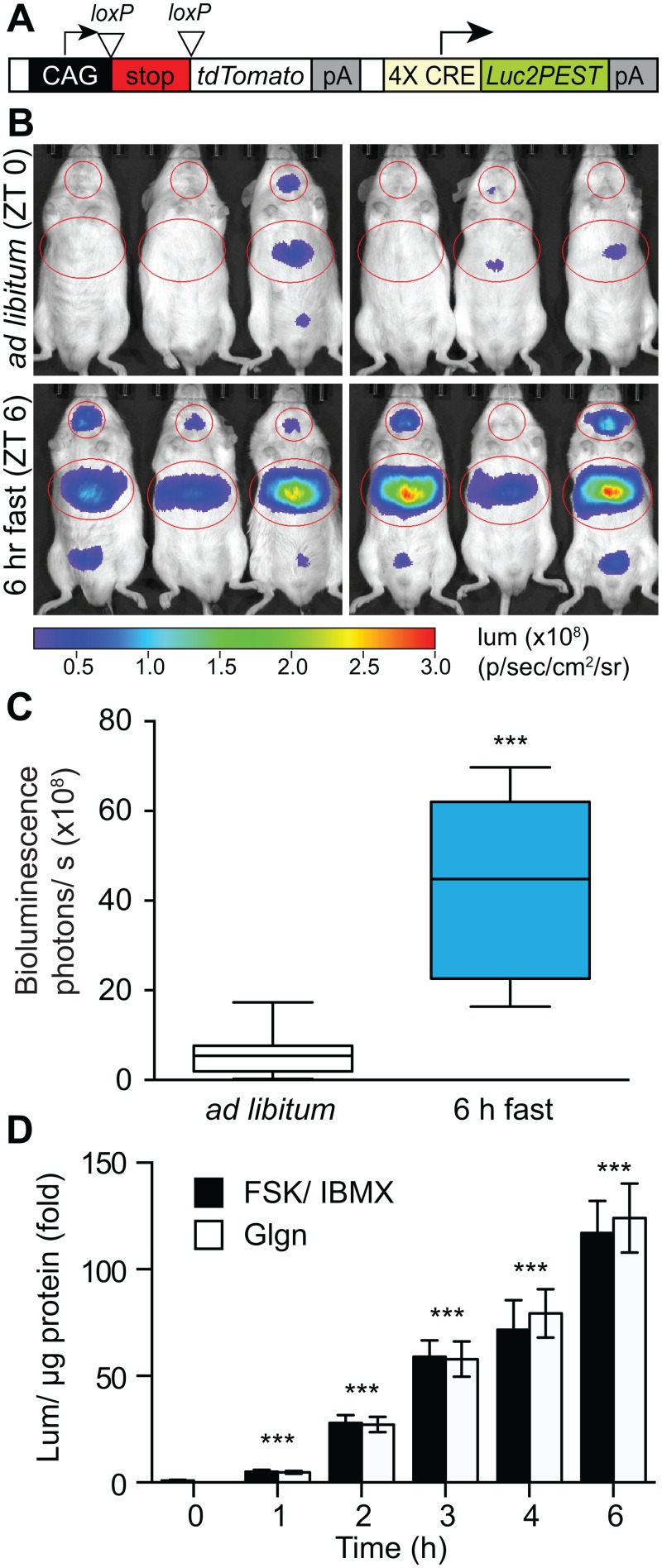
Validation of a *ROSA26-CRE-luc* knock-in mouse. (A) Schematic of *ROSA26-CRE-luc* knock-in construct. (B) Bioluminescence imaging of male *ROSA26-CRE-luc* knock-in mice, *ad libitum* fed (ZT0) and after a 6-h fast (ZT6) (5 sec exposure). (C) Quantification of hepatic bioluminescence in indicated region of interest in *ROSA26-CRE-luc* mice shown in B and four additional littermates (median, 25th and 75th percentile and range indicated, *n* = 10; ***, *p* = 0.002 by paired, 2-tailed *t*-test). (D) Luciferase activity in primary hepatocytes from *ROSA26-CRE-luc* knock-in mice treated with FSK/ IBMX or glucagon (Glgn) for indicated times. (*n* = 3 per time point; ***, *p*<0.001 to un-stimulated control). Panel D is representative of three independent experiments performed in triplicate.

To create *ROSA26* knock-in CREB reporter mice, we obtained a validated CREB-activated luciferase reporter plasmid from Promega that contains two full and two half cAMP response elements (CRE) and codon-optimized *luc2* fused to a destabilization sequence (PEST) (Fig 1A). We sub-cloned CRE-*luc2PEST* into the Ai9 *ROSA26* targeting vector, which also encodes a CAG-lox-stop-lox*-tdTomato* transgene that is commonly used as a *Cre* recombinase reporter [15]. The final allele retains the CAG-LSL-tdTomato cassette and can be simultaneously used as a *Cre* recombinase reporter when crossing to other tissue-specific knockout lines. We confirmed that tdTomato is expressed in primary hepatocytes from *ROSA26-CRE-luc* mice upon expression of *Cre* recombinase *in vitro* using an adenoviral vector (Ad-Cre) but not Ad-GFP control (S1A Fig). The CREB-activated luciferase transgene is not contingent upon *Cre* recombinase expression.

As expected, hepatic CRE-luciferase activity was low in *ad libitum* fed animals imaged at lights on (ZT0) and was markedly stimulated in the same animals imaged after fasting for 6 h (ZT6, Fig 1B and 1C). In addition to liver, we observed a trend (*p* = .10) toward increased luciferase signal from the brain after 6 h fasting (Figs 1B and S1B). We observed similar quantitative increases in hepatic CREB activity after overnight fasting from ZT8-day 2 ZT0 (S1C and S1D Fig). Hepatic bioluminescence signal further increased if animals were fasted for 16 hours, injected with the fasting hormone glucagon and imaged again 4 h later (S1E–S1G Fig). We confirmed that the CREB reporter is activated by fasting signals in hepatocytes by treating primary hepatocytes from *ROSA26-CRE-luc* mice with cAMP-inducing stimuli (FSK/IBMX or glucagon) (Fig 1D).

Hepatic CREB activity is known to be under circadian control [10]. In prior studies using an adenoviral CREB reporter, CREB activity was stimulated by 3 h fasting if animals were fasted at the transition from lights on to lights off (ZT10-13), but CREB was refractory to fasting at the transition from lights off to lights on ZT22- day 2 ZT1 due to increased expression of the transcriptional repressor *Cryptochrome1* at this time of day [10]. In our studies, hepatic CREB activity was generally low in *ad libitum* fed mice, whether tested at lights on (ZT0) or in the late afternoon (ZT8) and was stimulated in liver by fasting for 6 h during the day (starting at lights on, ZT0-ZT6, Fig 1B and 1C) or for 16 h fasting during the night (ZT8-day 2 ZT0, imaged at lights on, S1C and S1D Fig). The most likely reason we did not observe robust circadian regulation of fasting hepatic CREB-luciferase reporter signal is that we employed a longer duration of fasting than the prior study.

### *ROSA26-CRE-luc* mice reveal hepatic CREB activation after exercise

**Fig 2:**
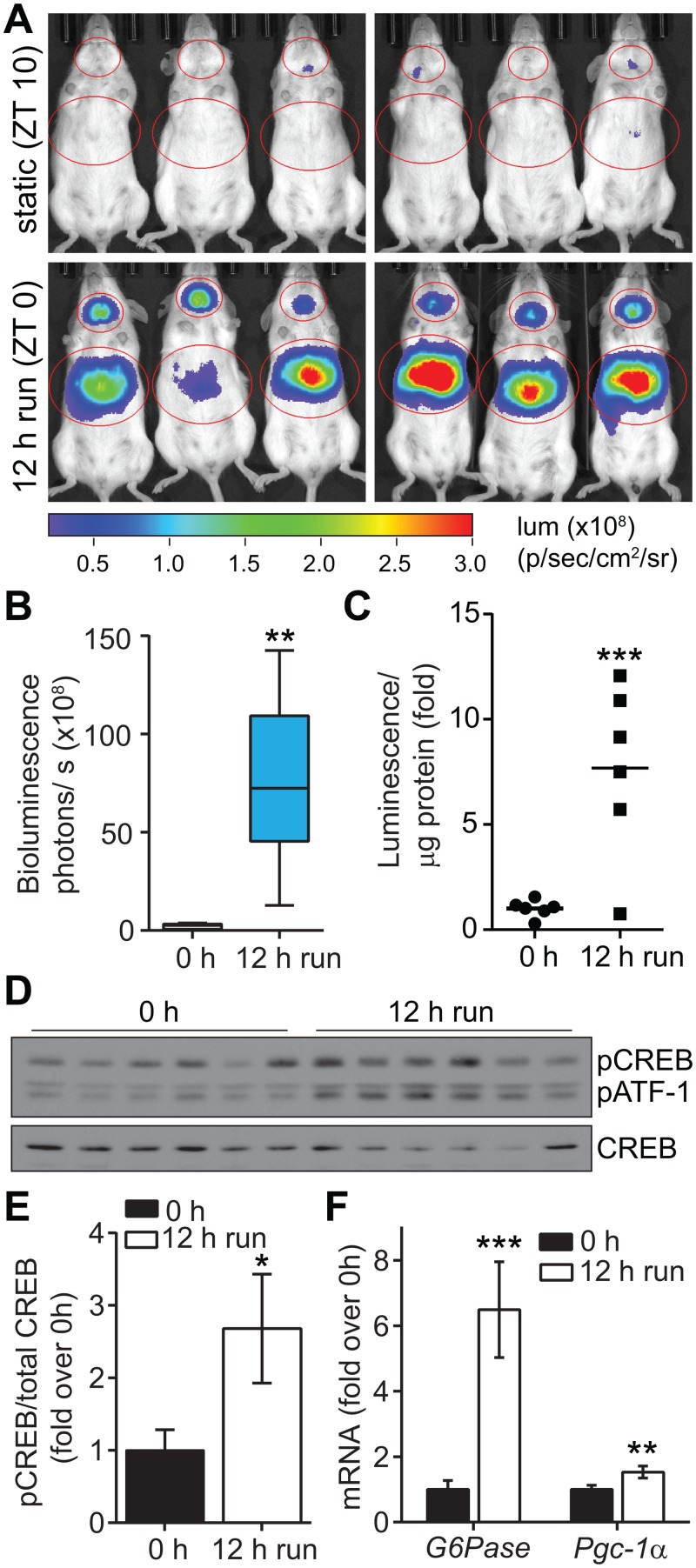
Hepatic CREB is activated after voluntary exercise. (A) Bioluminescence imaging of female *ROSA26-CRE-luc* knock-in mice (*n* = 6), static housed (0 h run, ZT10) and after 12 h voluntary wheel running exercise (ZT12-day2 ZT0) (5 sec exposure). (B) Quantification of hepatic bioluminescence in *ROSA26-CRE-luc* mice shown in A (*n* = 6, median, 25th and 75th percentile and range indicated). (C) Luciferase activity, (D) Western blot of phospho-CREB(S133)/ATF-1 and total CREB, (E) Quantification of pCREB western blots and (F) *G6Pase* and *PGC-1α* mRNA normalized to *Gapdh* from liver of mice run for 0 and 12 h (*n* = 6, mean ±SEM). *p<0.05, **p<0.01, ***p<0.001 by *t*-tests.

During exercise, glucose is rapidly utilized by skeletal muscle, creating a catabolic state. To compensate for glucose utilization and maintain glucose homeostasis during exercise, glucagon and catecholamines are released and, in turn, stimulate hepatic glucose production by glycogenolysis and gluconeogenesis [22, 23]. Liver expression of gluconeogenic genes encoding phosphoenol pyruvate carboxykinase (PEPCK) and glucose-6-phosphatase (G6Pase) increases within hours of running initiation in rodents [24–26]. As CREB is known to directly regulate transcription of *G6Pase* as well as the transcriptional regulator *Pgc-1α* in liver [2, 27, 28] and CREB phosphorylation in muscle has been shown to increase after 30 minutes of strenuous exercise [29], we tested CREB activity in female *ROSA26-CRE-luc* mice at baseline (static housing) and after 12 h of voluntary running during the dark cycle. We observed approximately 30-fold increased CREB reporter bioluminescence in liver of mice after 12 h voluntary running compared to the same mice measured before exercise (Fig 2A and 2B) and 10-fold increased bioluminescence emanating from the brain (Figs 2A and S2A). Accordingly, biochemical luciferase activity was increased in liver lysates of exercised mice compared with static housed controls (Figs 2C and S2B) and was correlated with hepatic *in vivo* bioluminescence signals (S2C Fig). CREB phosphorylation on Ser133, which is required for CREB activity [30], was increased about 2.5-fold in liver after 12 h of exercise (Fig 2D and 2E). Supporting these results, *G6Pase* and *PGC-1α* mRNAs were increased in liver of exercised mice relative to static housed controls (Fig 2F).

In contrast to liver, quadriceps muscle from the same mice did not show bioluminescence signals after 12 h voluntary running (Fig 2A). Interestingly, CREB phosphorylation was actually decreased in quadriceps and gastrocnemius muscle after 12 h of voluntary running compared with static controls (S2D–S2G Fig). We observed robust activation of the genomic CREB reporter by FSK/ IBMX treatment of primary myocytes from *ROSA26-CRE-luc* mice (S2H Fig), indicating that the artificial CREB reporter can be activated in cells of the muscle lineage. Together these and published data [29] indicate that CREB is likely transiently activated by exercise in skeletal muscle, but becomes down-regulated after prolonged duration of exercise. However, in liver, CREB activity is sustained after prolonged exercise.

## Discussion

We have generated CREB reporter mice and used these mice to monitor CREB activity in liver by *in vivo* bioluminescent imaging following fasting and voluntary exercise. The knock-in strategy that we used has several advantages over adenoviral vectors or randomly inserted transgenes [8, 14, 31]. First, adenoviral infection triggers an acute inflammatory response [32] and require co-infection with a control constitutive reporter adenovirus (such as RSV-β-galactosidase) for post-mortem normalization for infection efficiency [8]. In addition, adenovirus has highest tropism for liver, which limits its utility for other tissues, and transgene expression is acute, waning after approximately 2 weeks [13]. In contrast, *ROSA26-CRE-luc* mice allow for monitoring CREB activity longitudinally in a variety of physiological contexts. Finally, we observe minimal animal-to-animal variability when cohorts are generated from a single male breeder and subjected to a strong induction stimulus (*e*.*g*. S1G Fig, fasting plus glucagon).

We [14] and others [31] have previously created transgenic CREB-activated luciferase reporter mice by random genomic insertion using either the same CRE-containing promoter within a minimal promoter from the *CFTR* gene as in the adenoviral vector [14] or a promoter with six synthetic CRE sites in a HSV-TK (Herpes simplex virus-thymidine kinase) composite minimal promoter [31]. While both of these mouse lines have the advantage of germline transmission, disadvantages of random insertion yielded differential expression in different founder lines [31]. The animal line described here eliminates those disadvantages by having a single transgene gene in a known site in the genome that is known to have open chromatin [33]. Similar to the most recent transgenic [31], ours employs *Luc2*, which is codon-optimized for expression in mammalian cells. However, our line yields brighter bioluminescence signals, with approximately two orders of magnitude higher signal intensity in brains of our line after fasting compared with the former transgenic line after isoproterenol injection [31] imaged using similar instrument settings (*ROSA26-CRE-luc* brain signal ~2x10^7^ p/s/cm^2^/sr in 5 sec exposures; CRE-luc transgenic brain signal ~5x10^5^ p/s/cm^2^/sr in 1 min exposures). The two experimental paradigms differ, but the large difference in signal intensity may be due to transgene construction or genomic insertion site. An additional technical advance of the *ROSA26-CRE-luc* mouse line is the bi-functional nature of the *ROSA26*-based allele, which contains a *Cre* recombinase dependent *tdTomato* gene to report lineage *Cre* recombinase expression as well as CREB activity. The CREB-activated luciferase transgene is not dependent on *Cre* recombinase expression.

Similar to previous results using adenoviral CRE-luc [8, 9, 11] and qPCR to monitor CREB target gene expression [1, 2, 27], we observed low liver CREB activity in *ad libitum* fed mice and robust activation of CREB in liver after 6 h fasting, 16 h fasting or 16 h fasting followed by glucagon, as well as reporter activation in primary hepatocytes from *ROSA26-CRE-luc* mice treated with cAMP inducing ligands or glucagon *ex vivo*. Liver CREB activity was previously shown to be circadian regulated and refractory to fasting at the end of the dark cycle compared with fasting during the day [10]. We did not perform detailed circadian time courses with the shorter duration of fasting (3h), so it remains to be determined whether the *ROSA26-CRE-luc* strain will be useful for study of circadian regulation of CREB activity in brain and liver.

Bioluminescence increased in brains of *ROSA26-CRE-luc* mice, in catabolic conditions of fasting or exercise. This might in part reflect circadian regulation of cAMP levels and CREB activity in the brain, which rises steadily from ZT0 to a peak at ~ZT7 [34, 35]. However, we observed a specific induction under catabolic conditions, whereas *ad libitum* fed animals had little CREB activity in brain at either ZT0 or ZT8 [compare brain regions in Fig 1B (ZT0), Fig 2A (ZT10) and S1C Fig (ZT8)]. Our data are in keeping with reports showing elevated CREB phosphorylation in the hypothalamus after fasting [36, 37]. Interestingly, CREB and its target genes are also regulated in the hypothalamus in the post-prandial state: CREB and its co-activator CRTC1 are activated by a leptin-initiated circuit culminating in MC4R-dependent activation of CREB on the *Trh* (thyrotropin-releasing hormone) promoter and secretion of thyroid hormone to stimulate energy expenditure [38] as well as expression of *Cartpt* (*cocaine- and amphetamine-related transcript*) and *Kiss1* (*Kisspeptin*) to promote satiety [39]. Neuronal CREB activated by fasting most likely occurs in distinct neuronal types or nuclei from those activated in the post-prandial state.

Our data show for the first time that CREB is activated in liver in response to prolonged exercise. We observed strong bioluminescence, CREB phosphorylation and CREB target gene expression (*G6Pase* and *PGC-1α*) in liver following 12 hours of voluntary running. Catecholamines and glucagon are known to increase during exercise [40–43], and we speculate that hepatic CREB is stimulated as part of a physiologic response to low blood glucose during exercise to stimulate gluconeogenesis. Although this phenomenon has been most studied after an acute bout of treadmill running, our data are consistent with induction of *G6pase* mRNA in liver after treadmill exercise (1h or run to exhaustion) [24–26, 44].

We expected that, similar to treadmill exercise [29], long-term voluntary exercise would elicit sustained CREB phosphorylation in skeletal muscle, when CREB and its co-activators would contribute to muscle adaptation and remodeling via transcriptional induction of *Pgc-1α* [45, 46]. Strikingly we did not observe increased CREB phosphorylation after 12 h of voluntary running exercise. This was surprising, as CREB is activated by treadmill exercise and its family members directly bind to cAMP response elements in the *Pgc-1α* promoter in different cell types including hepatocytes and skeletal myocytes [2, 27, 46]. PGC-1α drives mitochondrial biogenesis and contributes to adaptation to exercise [47, 48], and *Pgc-1α* mRNA is increased in skeletal muscle after a 12-hour bout of running [49]. However, the CREB family protein ATF-2 has been shown to be activated by exercise and required for contraction-induced *Pgc-1α* promoter activation in skeletal muscle *in vivo* [50], pointing to the possibility that ATF-2 is the primary regulator of the cAMP response element in the *Pgc-1α* promoter in muscle in response to exercise. CREB phosphorylation in quadriceps and gastrocnemius muscle was actually reduced after 12 hours of voluntary wheel running exercise. We readily detected luciferase activity in *ROSA26-CRE-luc* primary myocytes exposed to cAMP-inducing agents, confirming that the reporter is functional in myogenic cells. We propose that CREB is rapidly activated in skeletal muscle after strenuous exercise, but feedback pathways limit CREB phosphorylation after longer durations of exercise. This model is consistent with the finding that expression of activated CREB caused little or no change in skeletal muscle *Pgc-1α* mRNA, mitochondrial activity, or exercise capacity [19]. It would be interesting to explore mechanisms that restrict CREB phosphorylation in skeletal muscle after longer times of exercise training.

## Conclusions

We have created a *ROSA26* knock-in CREB-activated luciferase reporter transgenic mouse line. These mice show robust hepatic CREB activity after fasting, exercise or administration of fasting hormones, and primary cells derived from these animals can be used to study molecular mechanisms of CREB regulation *in vitro*. This line affords highly sensitive luciferase imaging of CREB activity in liver and brain using a single transgene in a known genomic location and the opportunity for longitudinal studies of CREB activity in the same individual animals with other genetic mutations without using viral vectors. Using these mice, we have shown that the overall catabolic state after sustained voluntary wheel-running exercise profoundly activates CREB in liver, where it is necessary to drive gluconeogenesis. It will be exciting to use additional pharmacologic and physiologic challenges to test the utility of this line for monitoring dynamic CREB activity in other tissues and experimental paradigms.

## Supporting Information

S1 Fig Activation of the ROSA26-CRE-luciferase reporter by fasting. (A) tdTomato fluorescence in primary hepatocytes from *ROSA26-CRE-luc* mice infected with adenovirus encoding *Cre* recombinase (Ad-Cre) or GFP (Ad-GFP). Both viruses encode GFP. Scale bar = 100 μm. (B) Total bioluminescence (photons/sec) in brain ROI in images shown in Fig 1B and four additional mice (*n* = 10; *p* = 0.1 by Wilcoxon rank sum test). (C) Bioluminescence images on male heterozygous *ROSA26-CRE-luc* animals, *ad libitum* fed (ZT8) or fasted for 16 h (ZT8 through day2 ZT0). (D) Total bioluminescence in regions of interest (ROI) drawn over livers in C (*n* = 3, *p* = 0.055 by paired, 2-tailed *t*-test). (E) Diagram of fasting plus glucagon experiment in panels F and G. (F) Bioluminescence images on a separate set of male heterozygous *ROSA26-CRE-luc* animals fasted for 16 h (left, day 2 ZT0) followed by glucagon injection (100 μg/kg) and imaging 4 hours later right (day 2, ZT4). (G) Total bioluminescence in liver ROI of animals shown in F plus 4 additional littermates (*n* = 6, *p = .03 by paired Wilcoxon rank sum test).
(EPS)

S2 Fig Activation of CREB by running in brain and liver but not skeletal muscle. (A) Bioluminescence in brain of *ROSA26-CRE-luc* mice in running experiment shown in Fig 2A (*n* = 6, **p<0.01 by *t*-test). (B) Luciferase activity in liver lysates shown in Fig 2C (*n* = 6, mean ±SEM). (C) Correlation between bioluminescence shown in Fig 2A and 2B and luciferase activity shown in B. Pearson’s R coefficient = 0.8865. (D), (F) Western blot of phospho-CREB(S133)/ATF-1 and total CREB from quadriceps and gastrocnemius muscle of female *ROSA26-CRE-luc* knock-in mice shown in Fig 2A, static housed (0 h run, ZT10) and after 12 h voluntary wheel running exercise (ZT12-day2 ZT0). Proteins for probing with phospho-CREB and total CREB antibodies were run on two separate gels. (E), (G) Quantification of pCREB western blots shown in D and F. (H) Luciferase activity in primary myocytes from *ROSA26-CRE-luc* mice treated with FSK/ IBMX for 4 h (average of *n* = 3 independent experiments performed in triplicate, ** *p*<0.01 to un-stimulated control). **p<0.01, ***p<0.001 by *t*-tests.
(EPS)

S1 Table Oligonucleotide primer sequences used for gene targeting and qPCR. (PDF)

## Abbreviations

cAMP: cyclic adenosine monophosphate
CARTPT: cocaine- and amphetamine-regulated peptide
CBP: CREB binding protein
CFTR: cystic fibrosis transmembrane conductance regulator
CREB: cAMP response element binding protein
CRTC: CREB-regulated transcription coactivator
FSK: forskolin
IBMX: 3-isobutyl-1-methylxanthine
G6Pase: glucose-6-phosphatase
GLGN: glucagon
HSV: herpes simplex virus
KISS1: kisspeptin
LUC: luciferase (firefly)
PEPCK: phosphoenolpyruvate carboxykinase
PEST: peptide sequence rich in proline (P), glutamic acid (E), serine (S), and threonine (T)
PGC-1α: peroxisome proliferator-activated receptor-gamma coactivator
PKA: protein kinase A
TK: thymidine kinase
TRH: thyrotropin releasing hormone
ZT: zeitgeber time
