# Correction: Knock-in Luciferase Reporter Mice for *In Vivo* Monitoring of CREB Activity

**DOI:** 10.1371/journal.pone.0163576

**Published:** 2016-09-20

**Authors:** Dmitry Akhmedov, Kavitha Rajendran, Maria G. Mendoza-Rodriguez, Rebecca Berdeaux

In Supporting Information files S1 and S2 Figs, an extra un-lettered panel in the upper left corner was included in error. Please see the corrected S1 and S2 Figs here. The original lettered figure panels on S1 and S2 Figs and captions are correct. There is no change to the data or conclusions.

## Supporting Information

S1 Fig Activation of the ROSA26-CRE-luciferase reporter by fasting. (A) tdTomato fluorescence in primary hepatocytes from *ROSA26-CRE-luc* mice infected with adenovirus encoding *Cre* recombinase (Ad-Cre) or GFP (Ad-GFP). Both viruses encode GFP. Scale bar = 100 μm. (B) Total bioluminescence (photons/sec) in brain ROI in images shown in Fig 1B and four additional mice (n = 10; p = 0.1 by Wilcoxon rank sum test). (C) Bioluminescence images on male heterozygous *ROSA26-CRE-luc* animals, *ad libitum* fed (ZT8) or fasted for 16 h (ZT8 through day2 ZT0). (D) Total bioluminescence in regions of interest (ROI) drawn over livers in C (n = 3, p = 0.055 by paired, 2-tailed t-test). (E) Diagram of fasting plus glucagon experiment in panels F and G. (F) Bioluminescence images on a separate set of male heterozygous *ROSA26-CRE-luc* animals fasted for 16 h (left, day 2 ZT0) followed by glucagon injection (100 μg/kg) and imaging 4 hours later right (day 2, ZT4). (G) Total bioluminescence in liver ROI of animals shown in F plus 4 additional littermates (n = 6, *p = .03 by paired Wilcoxon rank sum test).
(EPS)

S2 Fig Activation of CREB by running in brain and liver but not skeletal muscle. (A) Bioluminescence in brain of *ROSA26-CRE-luc* mice in running experiment shown in Fig 2A (n = 6, **p<0.01 by t-test). (B) Luciferase activity in liver lysates shown in Fig 2C (n = 6, mean ±SEM). (C) Correlation between bioluminescence shown in Fig 2A and 2B and luciferase activity shown in B. Pearson’s R coefficient = 0.8865. (D), (F) Western blot of phospho-CREB(S133)/ATF-1 and total CREB from quadriceps and gastrocnemius muscle of female *ROSA26-CRE-luc* knock-in mice shown in Fig 2A, static housed (0 h run, ZT10) and after 12 h voluntary wheel running exercise (ZT12-day2 ZT0). Proteins for probing with phospho-CREB and total CREB antibodies were run on two separate gels. (E), (G) Quantification of pCREB western blots shown in D and F. (H) Luciferase activity in primary myocytes from *ROSA26-CRE-luc* mice treated with FSK/ IBMX for 4 h (average of n = 3 independent experiments performed in triplicate, ** p<0.01 to un-stimulated control). **p<0.01, ***p<0.001 by t-tests.
(EPS)
