# Supplementary material for: Knock-in Luciferase Reporter Mice for In Vivo Monitoring of CREB Activity
Source: PLoS One. 2016 Jun 23;11(6):e0158274. doi: 10.1371/journal.pone.0158274 (PMC4940169; doi:10.1371/journal.pone.0158274)
Supplement: S1 Table — (PDF) [file pone.0158274.s003.pdf]

Akhmedov, et al

S1 Table. Oligonucleotide sequences used

Oligo name	Sequence (5'-3')	Target
olg-ROSA-4F	GTAGGCAATACCCAGGCAAA	5'probe, 865bp
olg-ROSA-4R	GAGTCCCGATCCCCTACCTA	
olg-ROSA-2F	TGGCACTGTTCATTTGTGGT	3'probe, 779bp
olg-ROSA-2R	TTTGGATGGTTTTTGCATCA	
AI9-LUC-8	ATGCTGGGGAACTAGTGGTG	Sequencing
AI9-LUC-9	GTCGAAGATGTTGGGGTGTT	
AI9-LUC-10	ATGATCTGGTTGCCGAAGAT	
AI9-LUC-11	AACAGTACCGGATTGCCAAG	
AI9-LUC-12	GCGCCACCTTCTACTCCTC	
olg-ROSA-9F	CTCGAAGTACTCGGCGTAGG	genotype knock-in allele, 206bp [cit 21, Madisen et al]
olg-ROSA-9R	CTTGGCAATCCGGTACTGTT	
gt-tomato-wt-F	AAGGGAGCTGCAGTGGAGTA	genotype WT allele, 297bp [cit 21, Madisen et al]
gt-tomato-wt-R	CCGAAAATCTGTGGGAAGTC	
olq-mG6Pase-1F	TGCTGTGTCTGGTAGGCAAC	*G6Pase* mRNA
olq-mG6Pase-1R	AACATCGGAGTGACCTTTGG	
olq-mPGC1a-2F	GGACGGAAGCAATTTTTCAA	*Pgc-1α* mRNA
olq-mPGC1a-2R	TTACCTGCGCAAGCTTCTCT	
olq-mGAPDH-3F	AGGTCGGTGTGAACGGATTTG	*Gapdh* mRNA
olq-mGAPDH-3R	TGTAGACCATGTAGTTGAGGTCA	
